# The Red Blood Corpuscle

**Published:** 1870-06

**Authors:** 


***The Red Blood Corpuscle.***

At the recent reception of the American Medical Association, Surgeon Woodward showed the red blood corpuscle magnified 40,000 diameters. By a private letter, we are informed that he pointed out the central, elevated portion discovered by Prof. Freer, which was shown indistinctly. Referring to Prof. Freer's opinion that it was the nucleus, he remarked that he was himself inclined to the opinion that it was produced by drying and shrinking. Those who have seen (as we have repeatedly,) Prof. Freer's beautiful demonstrations from blood drawn freshly, and placed immediately on the slide, will at once appreciate the incorrectness of Surgeon Woodward's idea. By the way, the blood corpuscles furnished by renal hæmaturia, exhibit this central mammillated projection with remarkable clearness.

Speaking of the American Medical Association, our correspondent will pardon us for quoting and adopting the sentences with which he closes his note: "A great deal of time was wasted in the discussion of ethical questions. Until we can succeed in infusing—or transfusing—a little more honor into the veins of many medical men it will be useless to prate about ethics. And until we succeed in establishing medicine upon a more scientific basis, and meet together for scientific discussion, our meetings will be but of little avail, and '*that's what's the matter.*' "

